# Overexpression of *Thellungiella halophila* H^+^-pyrophosphatase Gene Improves Low Phosphate Tolerance in Maize

**DOI:** 10.1371/journal.pone.0043501

**Published:** 2012-08-30

**Authors:** Laming Pei, Jiemin Wang, Kunpeng Li, Yongjun Li, Bei Li, Feng Gao, Aifang Yang

**Affiliations:** School of Life Science, Shandong University, Jinan, Shandong, China; Kansas State University, United States of America

## Abstract

Low phosphate availability is a major constraint on plant growth and agricultural productivity. Engineering a crop with enhanced low phosphate tolerance by transgenic technique could be one way of alleviating agricultural losses due to phosphate deficiency. In this study, we reported that transgenic maize plants that overexpressed the *Thellungiella halophila* vacuolar H^+^-pyrophosphatase gene (*TsVP*) were more tolerant to phosphate deficit stress than the wild type. Under phosphate sufficient conditions, transgenic plants showed more vigorous root growth than the wild type. When phosphate deficit stress was imposed, they also developed more robust root systems than the wild type, this advantage facilitated phosphate uptake, which meant that transgenic plants accumulated more phosphorus. So the growth and development in the transgenic maize plants were not damaged as much as in the wild type plants under phosphate limitation. Overexpression of *TsVP* increased the expression of genes involved in auxin transport, which indicated that the development of larger root systems in transgenic plants might be due in part to enhanced auxin transport which controls developmental events in plants. Moreover, transgenic plants showed less reproductive development retardation and a higher grain yield per plant than the wild type plants when grown in a low phosphate soil. The phenotypes of transgenic maize plants suggested that the overexpression of *TsVP* led to larger root systems that allowed transgenic maize plants to take up more phosphate, which led to less injury and better performance than the wild type under phosphate deficiency conditions. This study describes a feasible strategy for improving low phosphate tolerance in maize and reducing agricultural losses caused by phosphate deficit stress.

## Introduction

Phosphorus (P) is one of the macronutrients that are essential for plant growth, development, and reproduction. It is a fundamental component that is present in major organic molecules, such as nucleic acids, ATP and membrane phospholipids, it also plays a central role in an array of metabolic processes such as energy transfer, cellular signal transduction and the regulation of many enzymes. Phosphate (Pi) is the predominant form of P that is most readily taken up and transported within the plant, however, the concentration of available Pi in soil is very low, typically 1–10 µM, which cannot satisfy the demand of plants [1], [2]. Consequently, Pi availability is frequently a limiting factor for crop production in many natural and agricultural ecosystems all over the world.

To cope with Pi limitation, plants have evolved a series of adaptive strategies to maintain internal Pi concentrations at levels that will support growth and reproduction. These responses include: remobilizing and conserving internal Pi and increasing acquisition of external Pi. Among the adaptive responses to low Pi stress, maintenance of root growth and expansion of root architecture are especially important for Pi acquisition because Pi is only taken up efficiently by root systems with large surface areas [3].

Maize (*Zea mays*) is an important grain and forage crop worldwide. Pi availability is critical in the early developmental stages of maize and therefore has an important effect on production [4]. However, maize is adversely affected by Pi deficiency in many areas where it is grown, particularly in the acid soils of tropical and subtropical regions and the calcareous soils of temperate regions. These soils account for more than half the area under maize cultivation [5]. The use of Pi-rich fertilizer can improve crop yields that are limited by Pi deficiency but this practice is costly and dangerous to aquatic ecosystems because of the resulting eutrophication. Therefore, engineering staple crops with improved tolerance to Pi deficiency by transgenic breeding technique is of significance to both agriculture and the environment.

Vacuolar H^+^-pyrophosphatase (V-H^+^-PPase) is an enzyme that maintains vacuolar pH and provides energy for tonoplast transport, is composed of a single polypeptide with a molecular mass of about 80 kDa and utilizes pyrophosphate (PPi) as substrate [6]. *TsVP* is a V-H^+^-PPase encoding gene cloned from *Thellungiella halophila*, the deduced translation product has similar characteristics to V-H^+^-PPases from other species, such as Arabidopsis and rice [7]. It has been shown that transgenic cotton plants overexpressing *TsVP* exhibited enhanced root growth compared to the wild type under both normal and salt stress conditions and were more resistant to salt stress than the wild type [8]. Li et al reported that transgenic maize plants overexpressing *TsVP* showed more robust root systems than the wild type under drought conditions and were more tolerant to drought stress [9]. Li et al. reported that in addition to maintaining vacuolar pH, the Arabidopsis H^+^-pyrophosphatase gene, *AVP1*, also controlled auxin - dependent development and that overexpression of *AVP1* in Arabidopsis led to larger root systems through the stimulation of auxin transport [10]. Park et al. reported that overexpression of *AVP1* in tomato plants enhanced root development which help confer water deficit stress resistance in transgenic plants [11]. Yang et al. reported that overexpression of *AVP1* in *Arabidopsis*, tomato and rice plants resulted in increased root growth and improved performance under low Pi stress compared to the wild type plants [12]. Pasapula et al. reported that the *AVP1*-expressing cotton plants showed more vigorous shoot and root growth than wild type plants under both of drought and salt stresses [13]. These findings suggested that the overexpression of vacuolar H^+^-pyrophosphatase gene could enhance abiotic stress tolerance and improve root growth in many plants. As the primary organ involved in the efficient uptake of all the mineral elements, more robust root systems can facilitate Pi uptake, thus relieving the damage caused by Pi deficiency. To test whether the vacuolar H^+^-pyrophosphatase gene could increase root growth in maize and improve tolerance to Pi deficiency, transgenic maize plants that overexpressed *TsVP* were created and tested for their performance under Pi deficit stress conditions.

In this study, the results showed that transgenic maize plants overexpressing *TsVP* were more tolerant than wild type to imposed Pi deficit stress. The more robust root systems suggested the explanation for the enhanced low phosphate tolerance in transgenic plants. Transgenic plants showed an increased expression of genes involved in auxin transport, which indicated that the development of larger root systems might due to enhanced auxin transport in transgenic plants. Furthermore, we presented data showing that the transgenic maize plants produced greater grain yield per plant under Pi deficiency than the wild type plants, which was the predicted agricultural value of this gene. This study showed that engineering root development in agriculturally important maize could help reduce plant damage and agricultural losses caused by phosphate deficient conditions.

## Results

### Molecular analysis of transgenic maize plants that overexpressed *TsVP*

**Figure 1:**
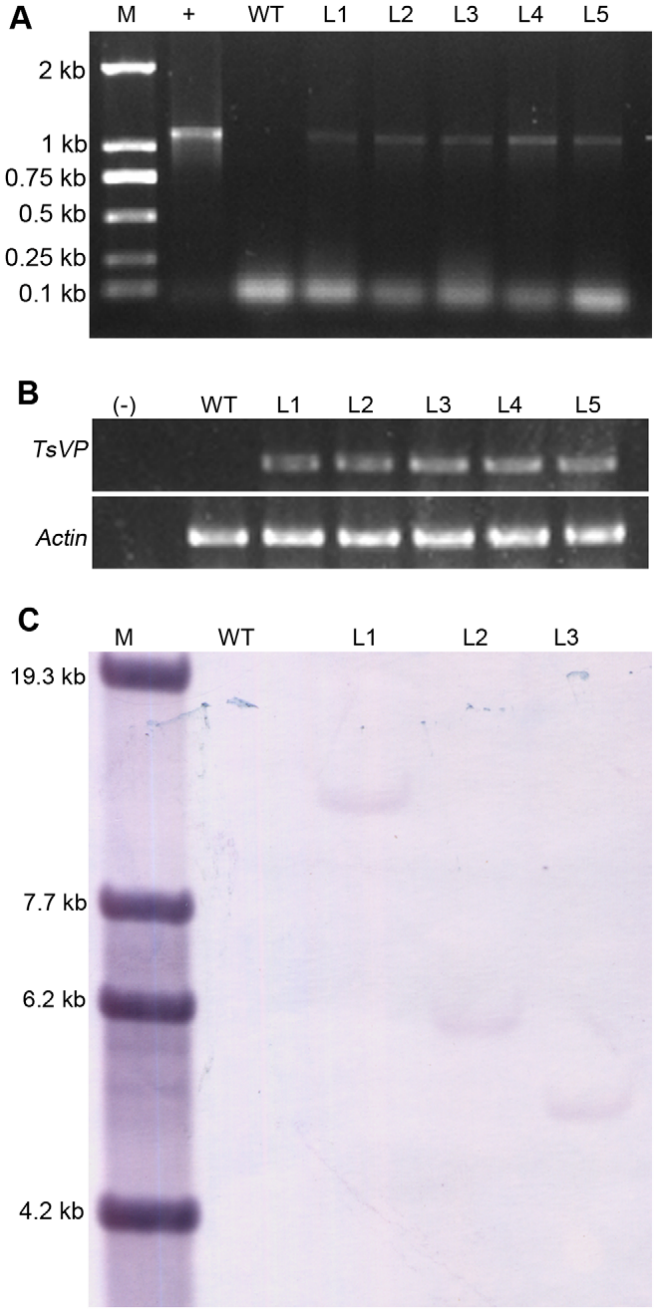
Molecular characterization of wild type and transgenic maize plants overexpressing *TsVP*. (A) polymerase chain reaction (PCR) analysis of wild type and transgenic lines with specific primers for *TsVP*. Lane M, DNA marker DL2000. Lane +, PCR result of plasmid pCAMBIA1300-Ubi-*TsVP-als*. Lane WT, wild type. Lanes L1 to L5, transgenic lines. (B) analysis of the expression of *TsVP* in wild type and transgenic lines by reverse transcription - polymerase chain reaction (RT-PCR) using maize actin1 as an internal control. Lane (−), H_2_O. Lane WT, wild type. Lanes L1 to L5, transgenic lines. (C) southern blot analysis of wild type and transgenic lines. Maize genomic DNA (50 µg) was digested with restriction enzyme *Bam*HI that contained no cut site in the *TsVP* cDNA region. A PCR - amplified fragment containing full - length *TsVP* cDNA was used as a probe. Lane M, *λDNA/EcoT14I* molecular weights. Lane WT, wild type. Lanes L1 to L3, transgenic lines.

Polymerase chain reaction (PCR) and reverse transcription-polymerase chain reaction (RT-PCR) analyses were performed on a wild type and five homozygous transgenic lines. The results indicated the presence and the expression of *TsVP* (Figure 1A and 1B). Three lines were selected for southern blot analysis, which indicated that they had a single T-DNA insertion (Figure 1C).

### Transgenic plants showed less growth retardation than the wild type plants under low Pi conditions

**Figure 2:**
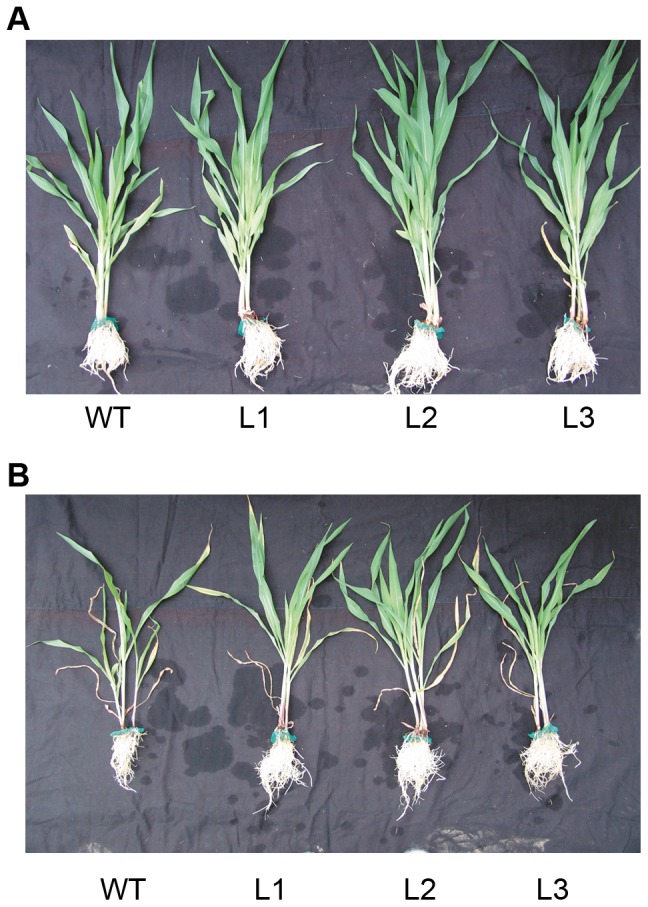
Wild type and transgenic maize plants. Maize seedlings were cultured in SP (1,000 µMKH_2_PO_4_) nutrient solution for 20 days, then divided into two groups and grown separately in SP and LP (5 µM KH_2_PO_4_) nutrient solutions for an additional 25 days. At this point, photographs were taken. (A) SP condition. (B) LP condition. WT, wild type; L1, L2 and L3, transgenic lines.

Maize seedlings were cultured in SP (1,000 µM KH_2_PO_4_) nutrient solution for 20 days, then divided into two groups and grown separately in SP and LP (5 µM KH_2_PO_4_) nutrient solutions for an additional 25 days. After 25 days of Pi deficit stress, all plants were adversely affected. However, the transgenic plants displayed less growth retardation than the wild type plants, which displayed significant growth inhibition (Figure 2).

### Transgenic plants had increased H^+^-PPase hydrolytic activity compared to the wild type plants

**Figure 3:**
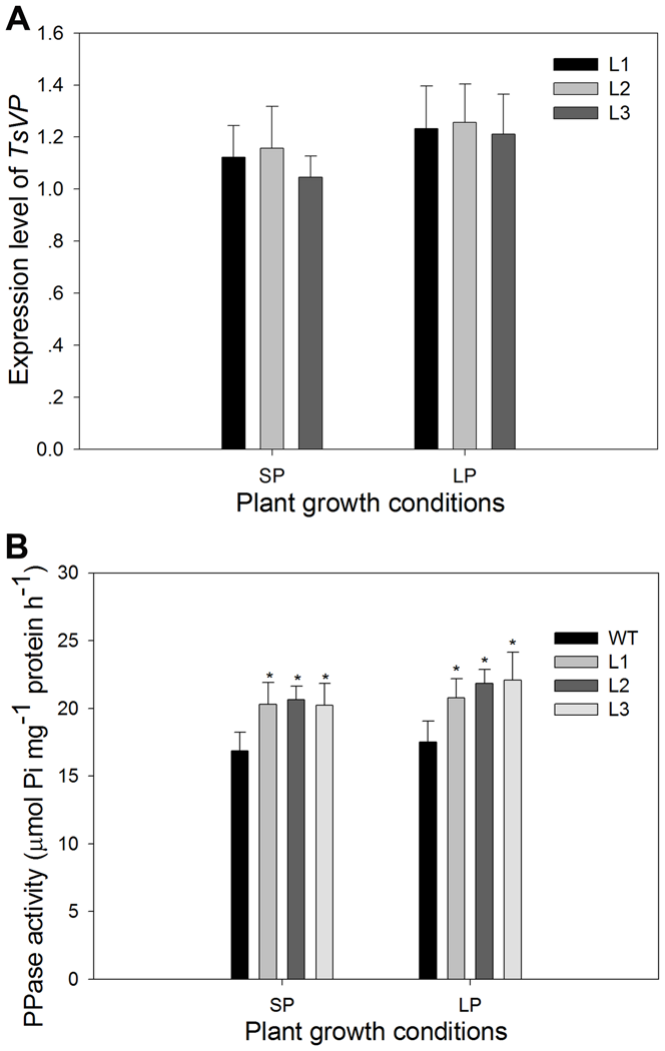
Relative expression of *TsVP* and H^+^-PPase activity in wild type and transgenic plants. The relative expression levels of *TsVP* (A) were shown as 2^−ΔΔCT^ [26], using maize actin1 as an internal control. The H^+^-PPase activity (B) was determined using root vacuolar membrane vesicles which were prepared by sucrose density gradient ultracentrifugation. The H^+^-PPase activity represented the K^+^-stimulated hydrolysis of the pyrophosphate (PPi) substrate. Values are means ± SD (*n* = 5). * indicates statistically significant differences between the transgenic lines and the wild type line under the same conditions at the 0.05 level, using Student's *t*-test. WT, wild type; L1, L2 and L3, transgenic lines; SP, sufficient phosphate (nutrient solution containing 1,000 µM KH_2_PO_4_); LP, low phosphate (nutrient solution containing 5 µM KH_2_PO_4_).

After 45 days of growth under hydroponic conditions as described above, the relative expression level of *TsVP* was examined by real-time RT-PCR in the root tips of maize plants. The results showed that *TsVP* was expressed in transgenic maize plants and there was no expression of *TsVP* in the wild type plants (Figure 3A). To confirm the functional expression of *TsVP*, vacuolar H^+^-PPase hydrolytic activity was determined using purified vacuolar membrane vesicles from roots of maize plants. As expected, transgenic plants showed higher (P<0.05) vacuolar H^+^-PPase activity than the wild type, both under SP and LP conditions (Figure 3B). Under SP conditions, transgenic plants had a mean 21% increase in vacuolar H^+^-PPase activity compared to the wild type plants; Under LP conditions, vacuolar H^+^-PPase activity levels were 20%, 21% and 24% higher in the L1, L2 and L3 plants compared to the wild type plants, respectively.

### Transgenic plants showed enhanced root growth compared to the wild type plants

**Figure 4:**
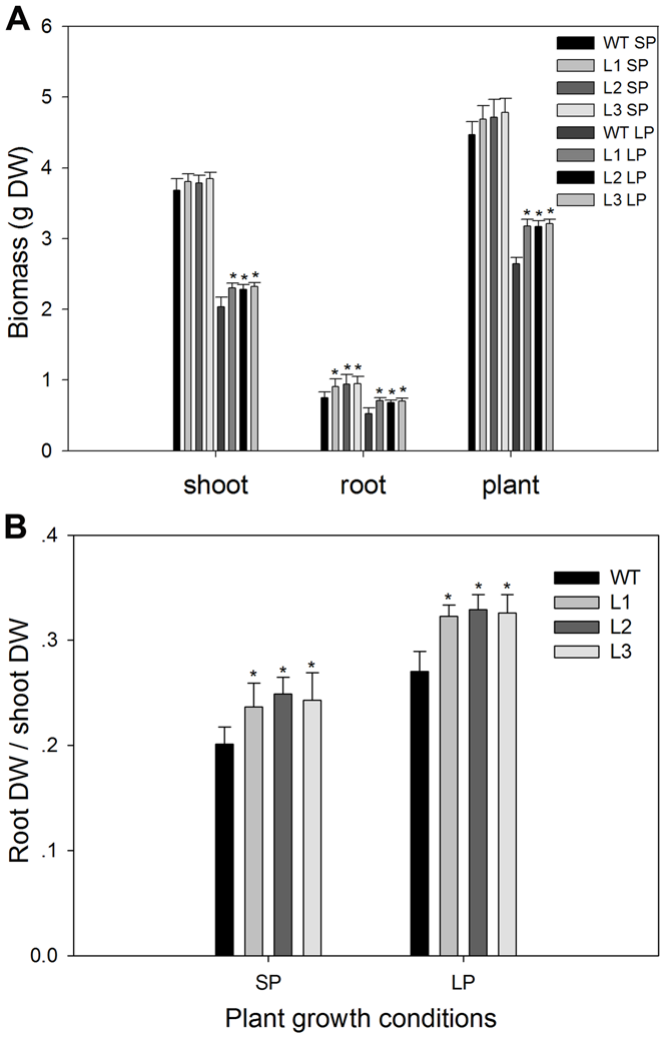
Biomass and root/shoot ratio in wild type and transgenic plants. (A) biomass. (B) root/shoot ratio. The dry weights of roots and shoots were determined after they were dried to constant weight in an oven at 80°C. Values are means ± SD (*n* = 5). * indicates statistically significant differences between the transgenic lines and the wild type line under the same conditions at the 0.05 level, using Student's *t*-test. DW, dry weight; WT, wild type; L1, L2 and L3, transgenic lines; SP, sufficient phosphate (nutrient solution containing 1,000 µM KH_2_PO_4_); LP, low phosphate (nutrient solution containing 5 µM KH_2_PO_4_).

Pi efficient maize genotypes usually have larger root systems with increased root dry biomass and root to shoot ratios [14], [15]. The biomass of the wild type and transgenic plants was measured after 45 days of growth in nutrient solution. As shown in Figure 4A, under SP conditions, transgenic plants had a mean 23% greater root biomass and showed no significant difference (P>0.05) in shoot biomass, compared to the wild type plants. Biomass accumulation in all plants was badly affected by low Pi stress, which was much less than that under SP conditions. However, transgenic plants were less affected. They (L1, L2 and L3) had 15%, 14% and 16% more shoot biomass, 40%, 37% and 39% more root biomass and 18%, 17% and 19% more plant biomass, respectively, than the wild type. As shown in Figure 4B, under both SP and LP conditions, the root dry weight to shoot dry weight ratio in transgenic plans was also significantly (P<0.05) higher than in the wild type. These data suggested that transgenic plants showed enhanced root growth under both SP and LP conditions and enhanced shoot growth under LP conditions, compared to wild type.

### Transgenic plants developed larger root systems than the wild type plants

After 45 days of growth in nutrient solution, maize plants were collected for root morphology measurement. As with the higher root biomass (Figure 4), transgenic plants were again found to have larger root systems than the wild type plants (Table 1). Under SP conditions, transgenic lines (L1, L2 and L3) had a mean 13% more axile roots, 11% more lateral roots, 14% longer total root length, 11% larger root volume, 12% larger root absorptive surface area, 15% more effective absorptive surface area per unit shoot dry weight and 12% longer average lateral root length compared to the wild type. Under SP conditions, all these parameters in transgenic plants were significantly different (P<0.05) from those in wild type. Under LP conditions, the number of axile and lateral roots, total root length, root volume, root surface area corresponding to unit shoot dry weight and average lateral root length of all the plants increased markedly, while average root diameter decreased significantly. This root morphology modification was an effective way of acclimatizing plants to the inadequate Pi supply by exploring more soil and acquiring more nutrients. Transgenic plants exhibited more significant changes. They had 30–34% more axile roots, 19–22% more lateral roots, 32–34% longer total root lengths, 17–19% larger root volumes, 29–31% larger root absorptive surface areas, 31–33% more effective absorptive surface area per unit shoot dry weight and 11–13% longer average lateral root lengths compared to the wild type plants, there were significant differences (P<0.05) in all parameters described above between transgenic plants and wild type under LP conditions.

**Table 1:** Root morphology of wild type and transgenic plants.

	SP				LP			
Item	WT	L1	L2	L3	WT	L1	L2	L3
Number of axile root g^−1^ DS	17.6±1.10	21.6±0.82*	20.9±0.52*	22.4±0.95*	21.5±1.34	28.7±1.96*	29.2±1.73*	29.4±1.69*
Number of lateral root g^−1^ DS	446±8.73	496±15.5*	495±12.1*	499±8.72*	515±29.8	610±43.7*	621±38.3*	626±37.7*
Total root lengh (m g^−1^DS)	4.55±0.21	5.31±0.19*	5.25±0.24*	5.27±0.16*	6.05±0.10	8.02±0.32*	8.11±0.29*	8.11±0.22*
Average root diameter (mm)	1.06±0.03	1.03±0.03	1.04±0.04	1.04±0.02	0.97±0.02	0.93±0.02	0.93±0.02	0.93±0.01
ALLR (mm)	7.12±0.03	8.10±0.09*	8.09±0.09*	8.08±0.08*	8.71±0.60	9.89±0.64*	9.81±0.46*	9.73±0.45*
Root volume (ml g^−1^ DS)	4.01±0.16	4.53±0.22*	4.48±0.20*	4.54±0.19*	4.45±0.20	5.30±0.27*	5.37±0.24*	5.40±0.23*
Root surface area (m^2^ g^−1^ DS)	0.75±0.02	0.88±0.03*	0.88±0.02*	0.89±0.03*	0.85±0.04	1.18±0.06*	1.19±0.06*	1.19±0.05*
EASA (m^2^ g^−1^ DS)	0.13±0.01	0.15±0.01*	0.15±0.01*	0.16±0.01*	0.05±0.01	0.08±0.01*	0.08±0.01*	0.08±0.01*
EASA %	17.00±1.46	17.10±1.50	17.10±1.93	17.21±1.63	6.40±0.74	6.75±0.47	6.71±0.84	6.70±0.59

Maize seedlings were cultured in SP (1,000 µMKH_2_PO_4_) nutrient solution for 20 days, then divided into two groups and grown separately in SP and LP (5 µM KH_2_PO_4_) nutrient solutions for an additional 25 days. DS, dry shoot; ALLR, average length of lateral roots and EASA, effective absorptive surface area. Values are means ± SD (*n* = 5).

* indicates statistically significant differences between transgenic lines and the wild type line under the same conditions at the 0.05 level, using Student's *t*-test. WT, wild type; L1, L2 and L3, transgenic lines; SP, sufficient phosphate (nutrient solution containing 1,000 µM KH_2_PO_4_); LP, low phosphate (nutrient solution containing 5 µM KH_2_PO_4_).

### Transgenic plants exhibited enhanced nutrient solution acidification compared to the wild type under Pi deficiency

**Figure 5:**
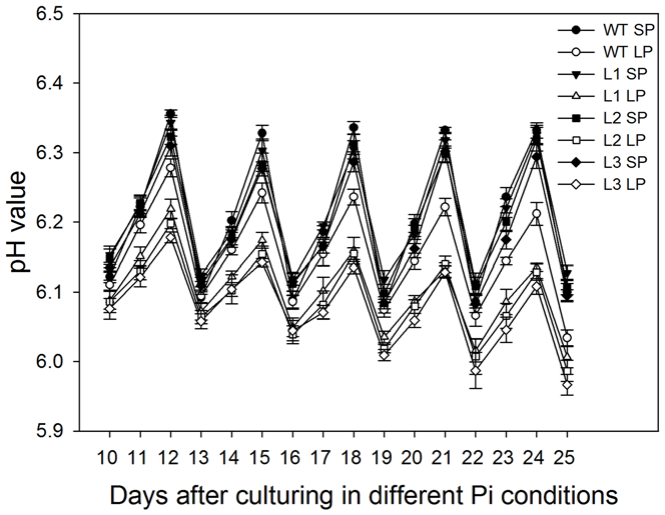
The pH value of nutrient solution in which maize plants were cultured. Maize seedlings were cultured in SP (1,000 µMKH_2_PO_4_) nutrient solution for 20 days, then divided into two groups and grown separately in SP and LP (5 µM KH_2_PO_4_) nutrient solutions for an additional 25 days. After 10 days of culturing in different Pi conditions, the pH value of nutrient solution was measured every day. Each of maize plants was cultured in 1 liter of nutrient solution. The initial pH value of nutrient solution was 6.0±0.1. Nutrient solution was replaced every 3 days. In this figure, on the 10th, 13th, 16th, 19th, 22th and 25th day of low Pi stress, the nutrient solution was replaced. Values are means ± SD (*n* = 5). WT, wild type; L1, L2 and L3, transgenic lines; SP, sufficient phosphate (nutrient solution containing 1,000 µM KH_2_PO_4_); LP, low phosphate (nutrient solution containing 5 µM KH_2_PO_4_).

One of adaptive response by plants to low Pi stress is increasing rhizosphere acidification capacity, which helps to increase displacement of Pi from rhizosphere insoluble complexes [16]. The larger root surface areas resulted from more robust root systems in transgenic plants (Table 1) would be expected to increase the acidification of nutrient solution. To test this hypothesis, after 10 days of low Pi stress, the pH value of nutrient solution was measured every day (Figure 5), each of maize plants was cultured in 1 liter of nutrient solution. Under SP conditions, there were no significant differences (P>0.05) in pH value of nutrient solution between transgenic plants and wild type. D:\Program Files\Youdao\Dict4\5.0.33.3225\resultui\queryresult.htmlUnder LP conditions, for all maize plants, the pH value of nutrient solution decreased obviously compared to that under SP conditions, the difference in the pH value between two Pi levels became increasingly obvious as low Pi stress time extended. Moreover, transgenic plants showed significantly lower (P<0.05) pH value of nutrient solution than wild type under Pi deficiency, which suggested that transgenic maize plants had a enhanced rhizosphere acidification compared to wild type.

### Transgenic plants showed faster Pi influx rates than the wild type plants

**Figure 6:**
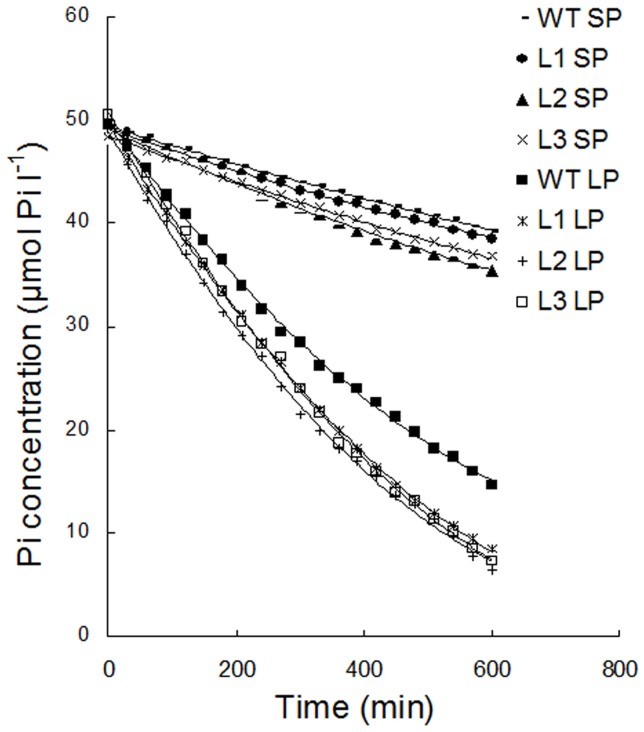
Depletion of Pi from a 500 ml nutrient solution over time by maize plants. Maize seedlings were cultured in SP (1,000 µM KH_2_PO_4)_ nutrient solution for 20 days, then divided into two groups and grown separately in SP and LP (5 µM KH_2_PO_4_) nutrient solutions for an additional 25 days. Then all maize plants were transferred to the nutrient solution that contained 50 µM Pi at the start. Pi uptake was measured as the amount of Pi removed from the nutrient solution. The points represent observed values. The curve was fitted using an expression based on Michaelis-Menten Kinetics. WT, wild type; L1, L2 and L3, transgenic lines; SP, sufficient phosphate (nutrient solution containing 1,000 µM KH_2_PO_4_); LP, low phosphate (nutrient solution containing 5 µM KH_2_PO_4_).

As the primary organ responsible for the efficient uptake of all the mineral elements required for plant growth and development, the more robust root systems (Table 1) seen in transgenic plants would be expected to increase the acquisition of Pi. To test this hypothesis, Pi uptake kinetics which reflected the Pi absorption status of the plants was measured (Figure 6 and Table 2). Maize seedlings were cultured in SP nutrient solution for 20 days, then divided into two groups and grown separately in SP and LP nutrient solutions for an additional 25 days. Then all maize plants were transferred to the nutrient solution that contained 50 µM Pi at the start. Pi uptake was measured as the amount of Pi removed from the nutrient solution. I_max_ represented the maximum rate of Pi influx. C_min_ represented the minimum concentration of solution below which no further net influx occurred. K_m_ reflected the affinity of Pi transporters for Pi. Under both SP and LP conditions, significant differences (P<0.05) in the I_max_ values between transgenic and wild type plants were observed. The I_max_ values of transgenic plants were 18–30% higher under SP conditions and 42–48% higher under LP conditions compared to the wild type plants. However, the C_min_ and K_m_ values for transgenic plants were not significantly different (P>0.05) from the wild type plants under both SP and LP conditions. These data indicated that transgenic maize plants showed no difference in transporter affinity for Pi compared to the wild type plants but showed faster Pi influx. The higher Pi influx rate might be due to the larger absorptive surface areas and the effective absorptive surface areas of the transgenic plants (Table 1), which were utilized to acquire Pi more efficiently.

**Table 2:** Pi uptake kinetics of wild type and transgenic plants.

Conditions	Lines	I_max_ (µmol l^−1^ h^−1^)	C_min_ (µmol l^−1^)	K_m_ (µmol l^−1^)
SP	WT	1.24±0.11	24.56±2.72	31.06±2.61
	L1	1.58±0.09*	26.25±2.66	33.46±2.72
	L2	1.67±0.13*	25.77±3.13	31.59±3.02
	L3	1.73±0.13*	24.66±2.88	31.53±2.46
LP	WT	3.52±0.25	4.21±0.41	17.03±1.03
	L1	5.06±0.24*	4.91±0.61	17.61±1.21
	L2	5.21±0.31*	4.41±0.56	16.20±0.84
	L3	5.10±0.28*	4.11±0.63	17.01±0.92

Maize seedlings were cultured in SP nutrient solution for 20 days, then divided into two groups and grown under SP and LP conditions for an additional 25 days. Then all maize plants were transferred to the nutrient solution that contained 50 µM Pi at the start. Pi uptake was measured as the amount of Pi removed from the nutrient solution. I_max_, maximum rate of Pi influx; C_min_, minimum concentration of solution below which no further net influx occurred and K_m_, Michaelis - Menten constant. Values are means ± SD (*n* = 3).

* indicates statistically significant differences between transgenic lines and the wild type line under the same conditions at the 0.05 level, using Student's *t*-test. WT, wild type; L1, L2 and L3, transgenic lines; SP, sufficient phosphate (nutrient solution containing 1,000 µM KH_2_PO_4_); LP, low phosphate (nutrient solution containing 5 µM KH_2_PO_4_).

### Transgenic plants accumulated more phosphorus than the wild type plants

**Figure 7:**
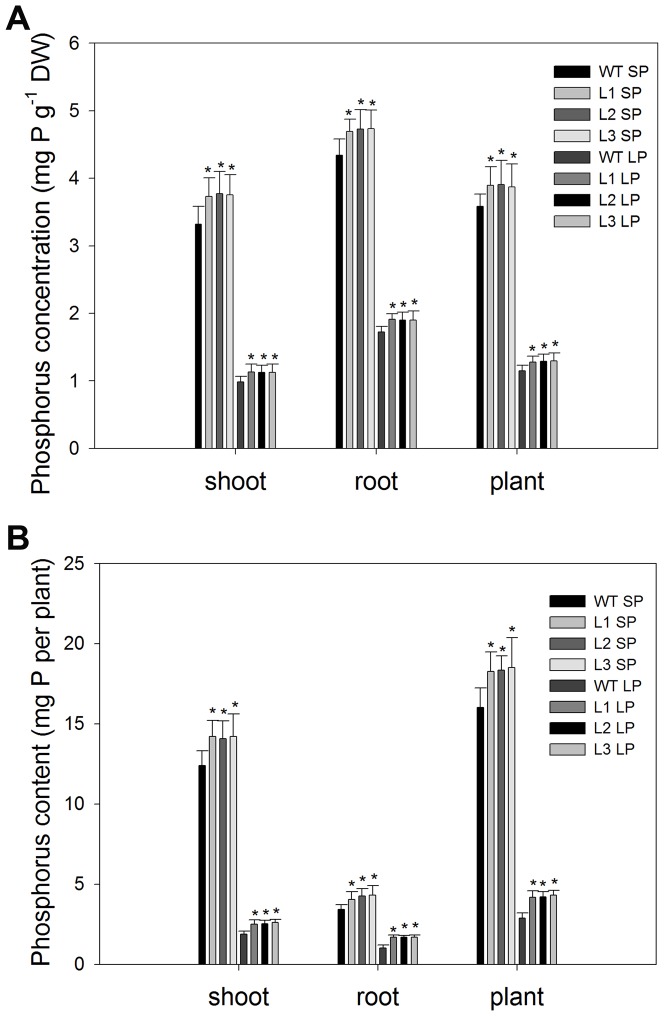
P concentration and P content in wild type and transgenic plants. (A) P concentration. (B) P content. Roots and shoots were digested using the H_2_SO_4_–H_2_O_2_ method. The P concentration was determined as described by Murphy and Riley [30]. Values are means ± SD (*n* = 5). * indicates statistically significant differences between transgenic lines and the wild type line under the same conditions at the 0.05 level, using Student's *t*-test. DW, dry weight. WT, wild type; L1, L2 and L3, transgenic lines; SP, sufficient phosphate (nutrient solution containing 1,000 µM KH_2_PO_4_); LP, low phosphate (nutrient solution containing 5 µM KH_2_PO_4_).

The more robust root systems (Table 1) and higher Pi influx rate (Figure 6 and Table 2) seen in transgenic plants were likely to result in a greater accumulation of P. To test this assumption, the P concentration (mg P/g dry weight) in the maize plants was determined (Figure 7A). Under SP conditions, the transgenic lines had a mean 14% higher shoot P concentration, 8.0% higher root P concentration and 12% higher plant P concentration compared to the wild type line. After 25 days of Pi deficit stress, all maize plants had a much lower P concentration than plants grown under SP conditions. However, the transgenic lines were less affected. They had a mean 15% higher shoot P concentration, 11% higher root P concentration and 14% higher plant P concentration compared to the wild type. Moreover, transgenic plants had a significantly higher P content (mg P) (Figure 7B). Under SP conditions, the transgenic lines had a mean 14% higher shoot P content, 25% higher root P content and 15% higher plant P content than the wild type line. Under LP conditions, the transgenic lines had a mean 35% higher shoot P content, 60% higher root P content and 45% higher plant P content than the wild type line. The maintenance of greater P concentrations in transgenic plants occurred concomitantly with enhanced root growth (Figure 4 and Table 1), which could lead to increased external Pi acquisition. The enhanced accumulation of Pi could help alleviate growth inhibition caused by Pi deficiency. Which might be the reason for that the shoot growth in transgenic maize plants was not inhibited as much as in the wild type by Pi deficiency, as indicated by the greater shoot biomass under LP conditions (Figure 4A).

### APase activity assay

Increasing APase activity is a universal adaptive response by plants to low Pi stress [16]. As shown in Table 3, under LP conditions, APase activity in young roots, old roots and old leaves of all plants were much higher than in maize plants grown under SP conditions, which indicated that when plants were exposed to Pi starvation, APase activity increased in senescent tissue and at effective absorption sites, which led to an acceleration in Pi recycling, thus alleviating the stress. However, whether under SP or LP conditions, APase activity of the corresponding areas in transgenic plants showed no significant differences (P>0.05) compared to the wild type plants. These data suggested that the APase activity was not the major reason for the differences in performance under Pi deficiency between transgenic and wild type plants.

**Table 3:** Intracellular APase activity in wild type and transgenic plants.

		APase activity (µmol NP mg^−1^ protein h^−1^)		
Conditions	Lines	Young root	Old root	Old leaf
SP	WT	54.80±6.30	51.70±6.74	41.52±4.73
	L1	56.53±7.76	48.82±5.98	42.48±4.72
	L2	53.69±7.75	52.51±7.28	41.95±4.65
	L3	52.21±5.91	48.11±7.68	45.28±5.31
LP	WT	118.35±7.44	130.22±8.32	51.70±8.79
	L1	116.73±6.07	138.02±7.71	54.05±7.39
	L2	120.28±5.87	132.45±8.98	51.77±9.65
	L3	112.24±6.86	129.14±7.71	54.07±8.97

Young root is a 1 cm segment taken from the root apex; Old root is a 1 cm segment taken from the base of the root; Old leaf is the second leaf from the base of a seedling. APase activities were determined using p-nitrophenol phosphate (p-NPP) as the substrate. Values are means ± SD (*n* = 5).

### Expression analysis of genes involved in auxin transport

**Figure 8:**
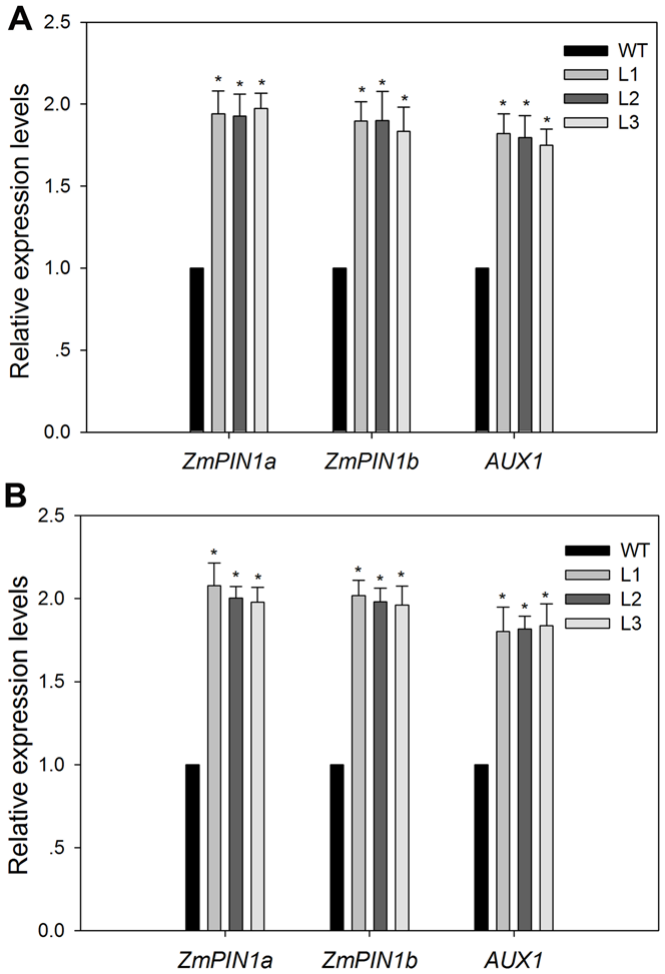
Relative expression levels of genes involved in auxin transport. The relative expression levels of of auxin transport genes were normalized to the wild type using maize actin1 as an internal control and (A) in sufficient phosphate (1,000 µM KH_2_PO_4_) nutrient solution. (B) in low phosphate (5 µM KH_2_PO_4_) nutrient solution. Values are means ± SD (*n* = 5). * indicates transgenic lines that are statistically different from the wild type line at the 0.05 level, using Student's *t*-test. WT, wild type; L1, L2 and L3, transgenic lines; SP, sufficient phosphate (nutrient solution containing 1,000 µM KH_2_PO_4_); LP, low phosphate (nutrient solution containing 5 µM KH_2_PO_4_).

Li et al. demonstrated that overexpression of *AVP1* in *Arabidopsis* resulted in more robust root systems than in the wild type line due to that AVP1 was able to facilitate auxin fluxes [10]. In order to study the correlation between the overexpression of *TsVP* and larger root systems in transgenic maize plants, the expression of some genes involved in auxin polar transport were analyzed by real-time RT-PCR at the root-shoot junction. *ZmPIN1a* (the accession numbers in the GenBank: DQ836239) and *ZmPIN1b* (the accession numbers in the GenBank: DQ836240) were auxin efflux carrier encoding genes in maize, *AUX1* (the accession numbers in the GenBank: AJ011794) encoded the AUX1 protein, an auxin influx carrier in maize. As shown in Figure 8, transgenic maize plants that overexpressed *TsVP* showed a higher relative expression level of these three genes compared to the wild type plants. This result suggested that enhanced root growth might be related to the increased auxin transport in transgenic plants.

### Transgenic maize plants showed less growth inhibition and reproductive development retardation than the wild type when grown in a low Pi soil

**Figure 9:**
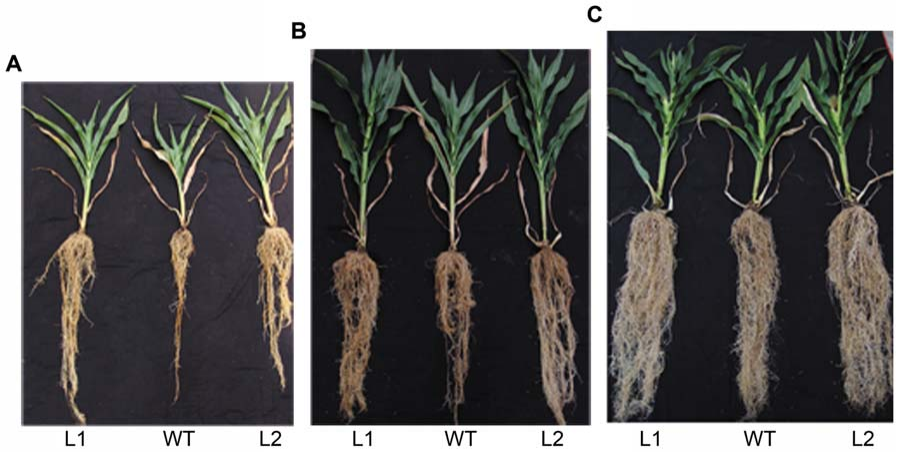
Wild type and transgenic maize plants at different growth stages. At the 40th (A), 60th (B), 80th (C) day of growth in low phosphate soil. The roots of maize plants were rinsed thoroughly under tap water and then photographs were taken. WT, wild type; L1, L2, transgenic lines.

To study the impact of Pi starvation on the final yield of wild type and transgenic maize plants that overexpressed *TsVP*, maize seeds were sown in flowerpots (diameter, 35 cm; height, 30 cm) containing a naturally low-Pi soil (10 mg Pi kg^−1^ soil) with either no additional KH_2_PO_4_ (low phosphate, LP) or mixed with 600 mg KH_2_PO_4_ (sufficient phosphate, SP) per kilogram soil and irrigated with tap water every two days until harvest. Plant growth and reproductive development in all plants were negatively influenced by Pi deficit stress. However, the transgenic plants showed less injury, they had significantly larger shoot and root systems (Figure 9) and less reproductive development retardation (Table 4). The reproductive development in the transgenic plants was not delayed as much as it was in the wild type plants. For the three transgenic lines, the growth times from seeding to flowering were 4.2, 4.1 and 3.7 days less, respectively, seeding to silking times were 5.7, 5.5 and 4.9 days less, respectively and ASI times were 1.6, 1.4 and 1.2 days less, respectively, compared to the wild type line, moreover, the increases in tassel branch number per plant were 31%, 34% and 44%, respectively, compared to the wild type (Table 4).

**Table 4:** ASI, number of tassel branches and the days taken to grow from seeding to various growth stages.

	SP				LP			
Item	WT	L1	L2	L3	WT	L1	L2	L3
Tasseling (days)	56.20±0.51	56.10±0.87	55.65±0.54	55.80±0.51	64.95±0.81	62.45±0.94*	62.55±0.54*	62.80±1.09*
Flowering (days)	61.05±0.48	60.10±0.62	59.75±0.77	60.05±0.94	72.50±0.58	68.30±0.73*	68.35±0.51*	68.70±0.54*
Pollen shedding (days)	61.30±0.52	60.30±0.41	60.20±0.44	60.75±0.58	73.75±0.58	69.30±0.74*	69.30±0.41*	69.75±0.58*
Silking (days)	61.90±0.84	60.70±1.08	60.35±0.83	60.50±0.51	76.20±1.01	70.50±0.91*	70.75±0.46*	71.35±0.37*
ASI (days)	0.85±0.06	0.60±0.08	0.60±0.05	0.45±0.08	3.70±0.44	2.10±0.37*	2.40±0.33*	2.64±0.37*
Tassel branches	9.60±1.51	10.60±1.14	10.00±1.00	10.60±1.14	5.80±0.44	7.60±0.54*	7.70±0.54*	8.40±0.54*

Maize seeds were sown in flowerpots containing a naturally low-Pi soil (10 mg Pi kg^−1^ soil) with either no additional KH_2_PO_4_ (low phosphate, LP) or mixed with 600 mg KH_2_PO_4_ (sufficient phosphate, SP) per kilogram soil. The days from seeding to tasseling, flowering, pollen shedding and silking were recorded. ASI was measured and tassel branches were counted. Values are means ± SD (*n* = 5).

* indicates statistically significant differences between transgenic lines and the wild type line under the same conditions at the 0.05 level, using Student's *t*-test. ASI, anthesis-silking interval; WT, wild type; L1, L2 and L3, transgenic lines; SP, sufficient phosphate and LP, low phosphate.

### Transgenic plants accumulated more biomass and phosphorus than the wild type plants when grown in a low Pi soil

**Figure 10:**
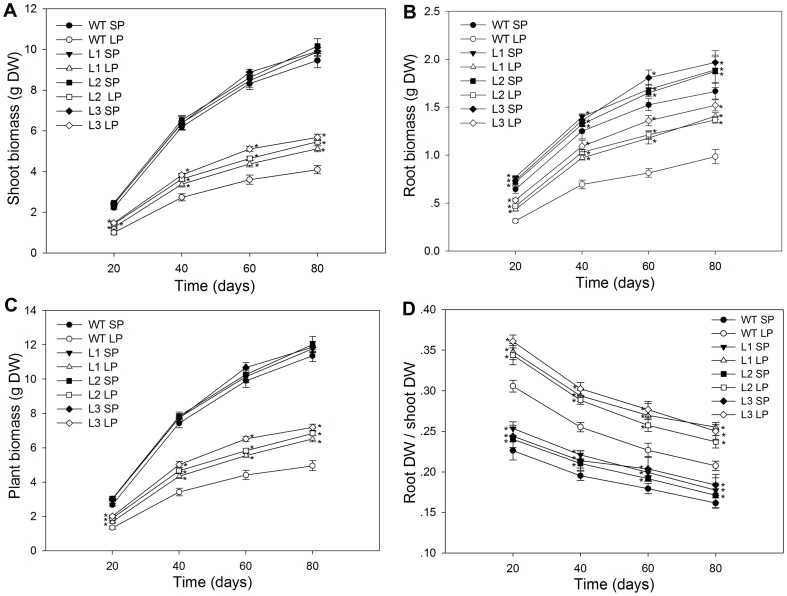
Biomass and root-shoot ratio of wild type and transgenic maize plants. Maize seeds were sown in flowerpots containing a naturally low-Pi soil (10 mg Pi kg^−1^ soil) with either no additional KH_2_PO_4_ (low phosphate, LP) or mixed with 600 mg KH_2_PO_4_ (sufficient phosphate, SP) per kilogram soil, one plant per flowerpot. On the 20th, 40th, 60th and 80th day of growth, the dry weights of wild type and transgenic plants were determined after they were dried to constant weight in an oven at 80°C. (A) shoot biomass. (B) root biomass. (C) plant biomass. (D) root dry weight-shoot dry weight ratio. Values are means ± SD (*n* = 5). DW, dry weight; WT, wild type; L1, L2 and L3, transgenic lines; SP, sufficient phosphate and LP, low phosphate.

**Figure 11:**
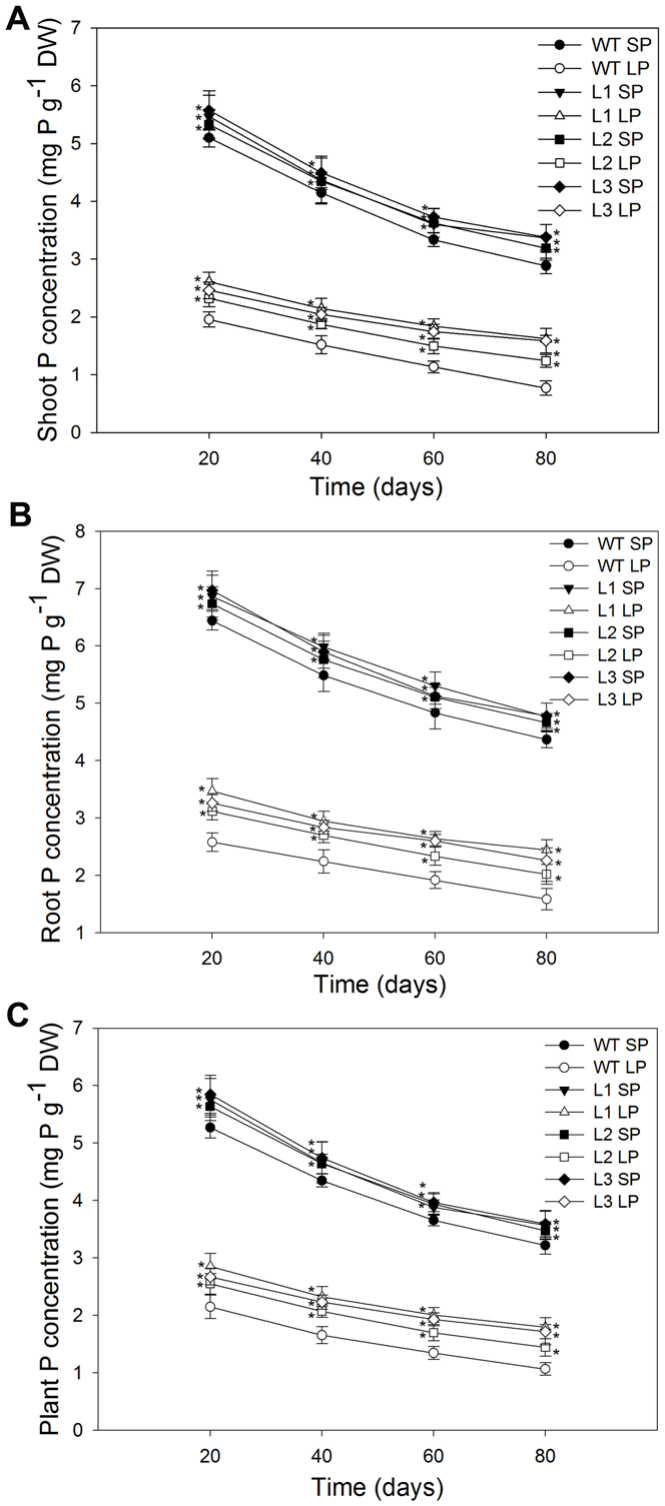
P concentration in wild type and transgenic maize plants. On the 20th, 40th, 60th and 80th day of growth in the flowerpots, roots and shoots from wild type and transgenic plants were digested using the H_2_SO_4_–H_2_O_2_ method in order to determine their P concentration. The P concentration was determined as described by Murphy and Riley [30]. (A) shoot P concentration. (B) root P concentration. (C) plant P concentration. Values are means ± SD (*n* = 5). P, phosphorus; WT, wild type; L1, L2 and L3, transgenic lines; SP, sufficient phosphate and LP, low phosphate.

**Figure 12:**
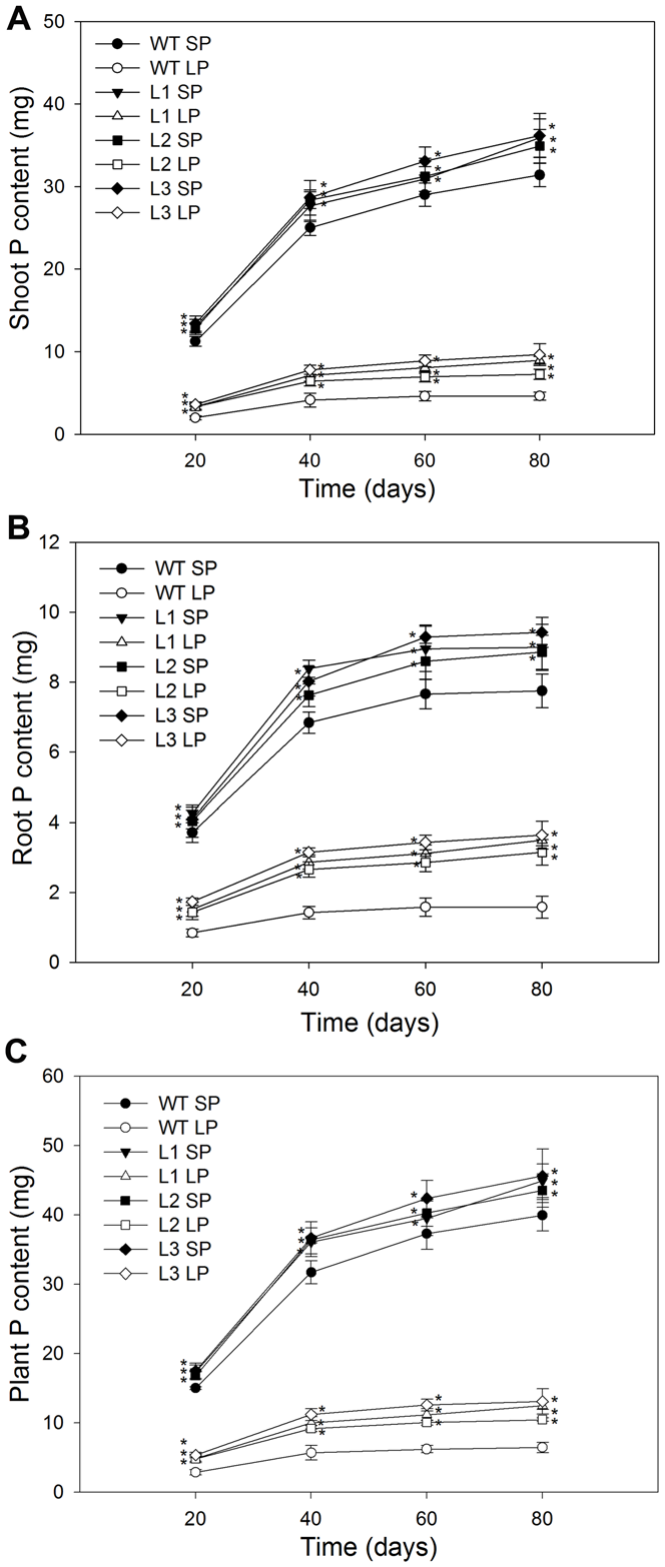
P content in wild type and transgenic maize plants grown in flowerpots. (A) shoot P content. (B) root P content. (C) plant P content. Values are means ± SD (*n* = 5). P, phosphorus; WT, wild type; L1, L2 and L3, transgenic lines; SP, sufficient phosphate and LP, low phosphate.

On days 20, 40, 60 and 80, the roots and shoots were excised to determine biomass (Figure 10), phosphorus concentration (mg P/g dry weight) (Figures 11) and phosphorus content (mg P) (Figures 12). Under SP conditions, transgenic plants had significantly higher (P<0.05) root biomass, phosphorus concentration and phosphorus content than the wild type. Under LP conditions, the differences between transgenic plants and the wild type were more notable, at four growth stages tested, the transgenic plants had a mean 51%, 52%, 56% and 51% higher root biomass, respectively (Figure 10B), 14%, 14%, 17% and 14% higher root to shoot ratio, respectively (Figure 10D). The more robust root (Table 1) and enhanced rhizosphere acidification (Figure 5) in transgenic plants would be expected to increase the accumulation of Pi. As expect, transgenic plants accumulated more phosphorus, at four growth stages tested, they had 21%, 32%, 45% and 60% higher shoot phosphorus concentrations, respectively (Figure 11A), 22%, 24%, 29% and 41% higher root phosphorus concentration, respectively (Figure 11B), 64%, 73%, 80% and 85% higher shoot phosphorus content, respectively (Figure 12A), 78%, 90%, 98% and 115% higher root phosphorus content, respectively (Figure 12B), compared to the wild type plants.

### Transgenic maize plants produced higher grain yield per plant than the wild type plants

**Figure 13:**
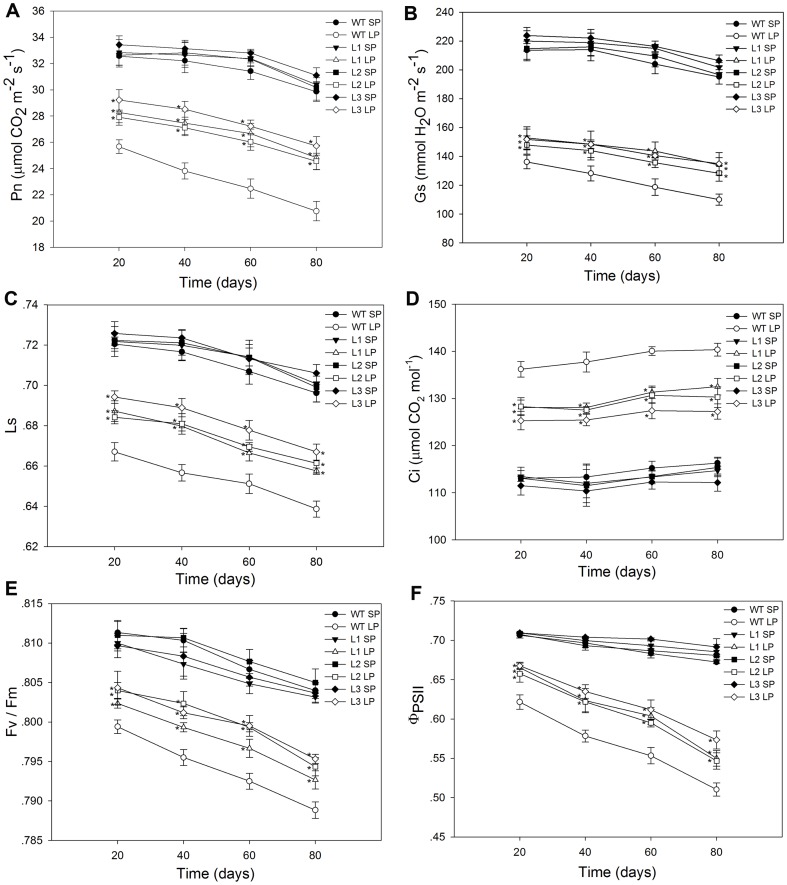
Photosynthetic performance of wild type and transgenic maize plants. On the 20th, 40th, 60th, 80th day of growth in the flowerpots, the gas exchange and chlorophyll fluorescence of wild type and transgenic maize plants were measured. (A) Pn (net photosynthesis). (B) Gs (stomatal conductance). (C) Ls (stomatal limitation). (D) Ci (intercellular CO_2_ concentration). (E) Fv/Fm (potential maximum photochemical efficiency of PSII). (F) *Φ*_PSII_ (actual quantum yield of PSII). Values are means ± SD (*n* = 5). PSII, photosystem II; WT, wild type; L1, L2 and L3, transgenic lines; SP, sufficient phosphate and LP, low phosphate.

**Figure 14:**
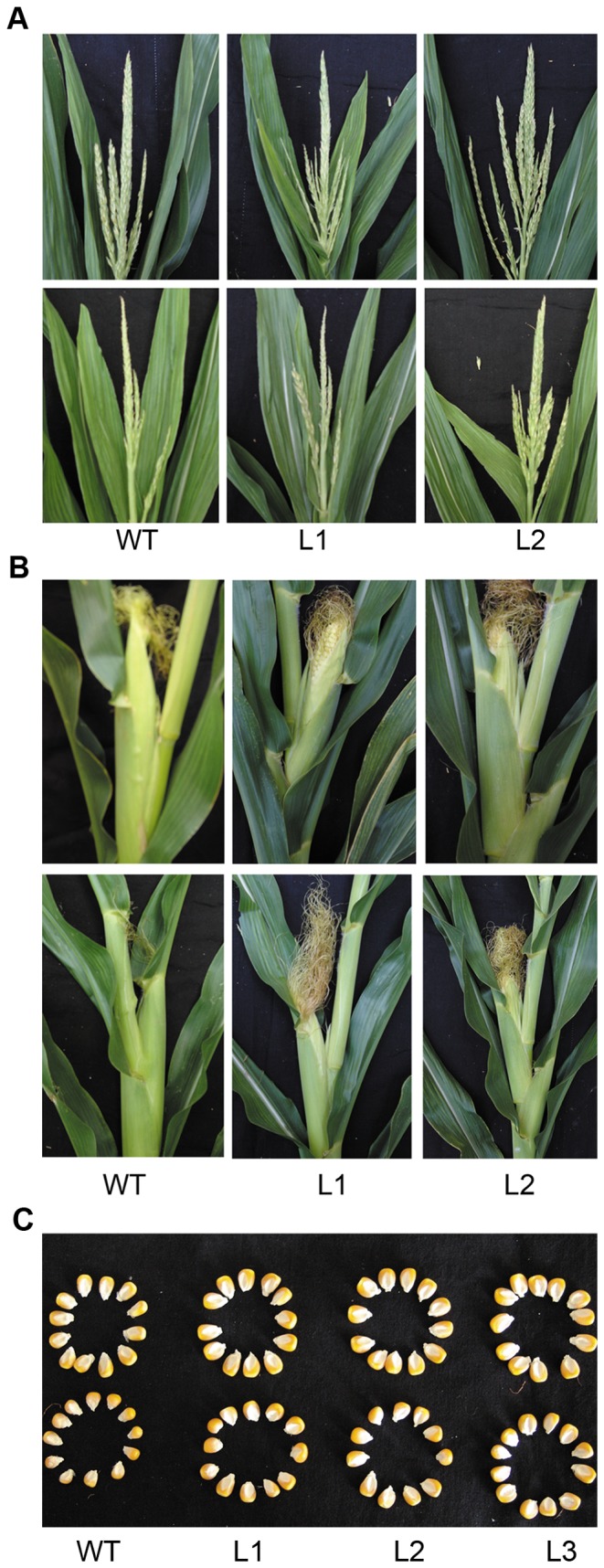
Tassels, Ears and kernels of wild type and transgenic maize plants. Transgenic plants had more tassel branches (A), larger ears (B) and kernels (C) than the wild type plants under LP conditions. WT, wild type; L1, L2 and L3 transgenic lines; SP, sufficient phosphate and LP, low phosphate.

Moreover, when grown in a low phosphate soil, net photosynthetic rates, Fv/Fm and *Φ*_PSII_ were significantly higher in transgenic plants than in the wild type plants (Figure 13). The reason might be that the increased phosphorus accumulation in transgenic plants (Figures 11 and 12) could relieve the inhibitory effects of Pi deficit stress on photosynthesis. Possibly because of the higher photosynthetic capacity and greater phosphorus accumulation, transgenic plants showed more shoot biomass (Figure 10A), larger ears and kernals (Figure 14) and greater grain yield per plant (Table 5) than the wild type plants under Pi stress conditions. For the three transgenic lines, the increases in grain yield per plant were 23%, 27% and 29%, respectively, compared to the wild type line. The higher grain yield per plant coincided with more kernels per row and heavier 1000-grain weights in the transgenic maize plants (Table 5).

**Table 5:** Wild type and transgenic maize yields from plants grown in flowerpots.

	SP				LP			
Item	WT	L1	L2	L3	WT	L1	L2	L3
Ear length (cm)	12.54±0.72	13.06±0.58	13.06±0.98	12.84±0.58	8.07±0.24	9.31±0.29*	9.98±0.31*	10.00±0.27*
Grain rows	12.80±1.09	12.80±1.09	13.20±1.09	13.20±1.09	12.00±0.02	12.00±0.02	12.00±0.02	12.00±0.02
Grain number per row	26.00±1.22	26.60±0.89	26.60±0.89	26.20±1.30	19.40±0.5	22.00±1.41*	23.00±1.58*	23.00±1.41*
Ear weight (g)	92.01±2.28	94.36±2.67	96.82±1.15	95.84±2.18	36.75±1.77	44.83±1.63*	47.88±2.21*	46.30±2.26*
Grain yield per plant (g)	81.66±1.84	83.91±1.07	86.55±1.64	84.94±1.54	32.18±1.46	40.10±1.63*	43.18±1.98*	41.70±1.26*
1000 grain weight (g)	244.9±3.49	246.1±3.49	244.9±4.26	245.7±3.58	133.1±4.5	151.1±3.21*	157.1±3.22*	151.2±3.15*

Maize seeds were sown in flowerpots containing a naturally low-Pi soil (10 mg Pi kg^−1^ soil) with either no additional KH_2_PO_4_ (low phosphate, LP) or mixed with 600 mg KH_2_PO_4_ (sufficient phosphate, SP) per kilogram soil. At harvest time, the ear length, number of grain rows and grain number per row of mature ears were recorded. Mature ears were dried and weighed to determine the grain yield per plant. Values are means ± SD (*n* = 5).

* indicates statistically significant differences between transgenic lines and the wild type line under the same conditions at the 0.05 level, using Student's *t*-test. WT, wild type; L1, L2 and L3, transgenic lines; SP, sufficient phosphate and LP, low phosphate..

## Discussion

Low phosphate availability is a major constraint on plant growth and agricultural productivity. Transgenic breeding through gene transfer is a method that can be able to create new corn varieties with enhanced tolerance to phosphate starvation. There are many reports on increased drought and salt tolerance in agriculturally important plants [9], [13]. However, as to improving low phosphate tolerance, most studies were performed with the model plants, little information is available nowadays about enhancing low phosphate resistance in maize.

The results of this study showed that overexpression of the *Thellungiella halophila* vacuolar H^+^-pyrophosphatase gene, *TsVP*, in transgenic maize plants resulted in low phosphate tolerance at several developmental stages. The overexpression of *TsVP* in maize plants increased root development, shoot growth, Pi uptake, phosphorus accumulation, photosynthetic performance and grain yield per plant compared to the wild type plants under Pi stress. This study has demonstrated that the development of a more robust root system in transgenic maize plants that overexpressed *TsVP* provided the morphological and/or physiological basis for these enhanced performances under Pi deficiency.

More extensive and robust root systems are usually associated with increased tolerance of maize to Pi deficit stress [14], [15], since large root systems have expanded root surface – soil contact areas and can explore larger volumes of soil, which are especially important in Pi acquisition because of low Pi mobility in soil. In this study, transgenic maize plants had higher root dry weights and root-shoot ratios (Figure 4, 10B and 10D), developed more extensive root systems (Table 1), both under Pi sufficient conditions and under Pi deficient conditions. Under normal growth conditions, the enhanced root growth was also seen in transgenic cotton plants that overexpressed *TsVP* [8]. Li et al. reported that changing *AVP1* expression in *Arabidopsis* resulted in development variations typical of alterations in auxin transport which controls developmental events in plants. Overexpression of *AVP1* increased the number and size of rosette leaves and enhanced root growth and dry weight in Arabidopsis, while homozygous *avp1* loss-of function mutant showed disrupted root and shoot development. The auxin transport and distribution rather than the total free auxin levels were affected by alterations in *AVP1* expression. The further discovery was that the P-ATPase-mediated driving forces for auxin transport and the expression of Pinformed 1 (PIN1) component of the auxin carrier were affected by alterations in *AVP1* expression. Thus, they concluded that overexpression of *AVP1* in *Arabidopsis* facilitated auxin transport by regulating the distribution and abundance of P-ATPase and PIN1, and consequently resulted in enhanced auxin-dependent root and shoot development [10]. In this study, the expressions of genes involved in auxin transport were analyzed (Figure 8). The results indicated that the relative expression level of these auxin transport gene were affected by overexpression of *TsVP*, which suggested that the enhanced root development in *TsVP*-overexpressing maize plants might also result from the enhanced auxin transport but further research is needed to confirm this. In this study, these auxin transport gene expressing pattern did not change drastically under LP conditions compared to SP conditions (Figure 8). It might be because that the expression of these auxin transport gene was not affected by Pi levels, but by the expression level of *TsVP* which had no obvious difference between LP conditions and SP conditions for 25 days (Figure 3).

As the primary organ responsible for the uptake of all the mineral elements required for plant growth and development, the more extensive root system allowed increased external Pi uptake, which was consistent with the I_max_ results, transgenic maize plants had higher I_max_ values than the wild type plants, both under SP conditions and under LP conditions (Figure 6 and Table 2). Pi is actively transported into the root by combining Pi transport proteins [17]. Some studies indicated that there were mainly two kinds of Pi transporters in plant roots, described as high-affinity and low-affinity systems, respectively. The low-affinity system was the system normally expressed in roots, whereas the high-affinity system was induced under low Pi stress conditions in order to increase Pi uptake [18], [19]. Data from this study confirmed these findings (Table 2). However, the C_min_ and K_m_ values in transgenic plants showed no significant differences (P>0.05) from the wild type plants (Table 2) both under SP and under LP conditions, which indicated that there was no difference in the affinity of Pi transporters for Pi between transgenic plants and wild type plants. Therefore, it could be concluded that the higher I_max_ values seen in transgenic plants were not due to the difference in the affinity of Pi transporters for Pi. Recent studies into Pi stress-induced gene expression showed that the number of Pi transporters partially determined the rate of Pi uptake [18]. The higher I_max_ values seen in transgenic plants could be due to greater numbers of Pi transporters resulting from the larger absorptive surface area (Table 1). The higher Pi influx rate (Table 2 and Figure 6) seen in transgenic plants were likely to result in a greater accumulation of P. Consistent with which, transgenic maize plants accumulated greater amount of P in shoots and roots than the wild type plants (Figure 7).

APase is thought to play a central role in the recycling of Pi to alleviate low Pi stress and its activity increased strongly in senescent tissue and at effective absorption sites when plants were exposed to low Pi stress [16], [20]. This was confirmed by these results (Table 3). However, whether under Pi sufficiency or Pi deficiency conditions, APase activity in the transgenic plants showed no significant difference (P>0.05) from the wild type plants, which suggested that the less growth retardation under Pi deficit stress (Figure 2) and enhanced performance displayed by transgenic plants had no correlation with the differences in APase activity.

In addition to the enhanced low Pi tolerance compared to the wild type when cultured under hydroponic conditions, maize plants that overexpressed *TsVP* also showed improved performance compared to the wild type plants when grown in low Pi soil. The transgenic plants also showed improved root growth (Figure 9 and 10) under both normal conditions and low Pi conditions. One of the most important physiological effects of Pi stress on plant metabolism is the reduction in photosynthetic capacity [21], [22], and the results of this study also found that in all maize plants grown in Pi deficient soil, photosynthetic rates, potential maximum photochemical efficiency of PSII (Fv/Fm) and actual quantum yield of PSII (*Φ*_PSII_) decreased significantly compared to plants grown in Pi sufficient soil (Figures 13 A, 13B, 13E and 13F). Pi plays a major role in the ATP cycle in both the light and dark reactions of photosynthesis and in the regulation of specific enzymes involved in carbon metabolism [23], so it was not surprising that photosynthetic capacity was inhibited by Pi deficiency. Farquhar et al. reported that the decrease in photosynthetic capacity was mainly due to stomatal or non-stomatal factors [24]. Jacob et al. reported that photosynthetic rate and stomatal conductance were reduced by Pi stress in maize but stomatal function was unaffected [21]. Consistent with these findings, in this study, the stomatal conductance value and stomatal limitation were smaller while the intercellular CO_2_ concentration was higher in maize grown under LP conditions compared to maize grown under SP conditions (Figures 13B, 13C and 13D), which indicated that the reduction in photosynthetic capacity was not mainly due to stomatal factors but, instead, was mainly due to non-stomatal factors. In this study, under low Pi conditions, photosynthetic rates, Fv/Fm and *Φ*_PSII_ values were higher in transgenic plants than in wild type plants (Figures 13A, 13E and 13F). The higher photosynthetic capacity was probably due to that greater phosphorus accumulation (Figures 11 and 12) in transgenic plants could help relieve the inhibitory effects of Pi deficit stress on photosynthetic capacity. Possibly because of the higher photosynthetic capacity and greater phosphorus accumulation, transgenic plants accumulated more shoot biomass (Figure 10A) and, most importantly, they produced higher grain yield per plant (Table 5) than wild type plants, which strongly suggested that *TsVP* could be used to alleviate agricultural losses from maize grown in low Pi soils. In addition to our findings, Yang et al has reported that overexpresion *AVP1* that encoded *Arabidopsis* vacuolar H^+^-pyrophosphatase in tomato resulted in higher fruit production in Pi deficient soils [12].

In summary, the results showed that the overexpression of *TsVP* in maize plants improved root system development and this phenotype can help confer a higher degree of tolerance to phosphate stress. This approach should be applicable to breeding of new maize varieties with improved low phosphate resistance. This research indicated that the *TsVP* gene has the potential to be used for improving crop's low phosphate tolerance and yields in areas where low Pi availability is a limiting factor for agricultural productivity.

## Materials and Methods

### Plant material

The maize inbred line, DH4866 (wild type, WT) and its transgenic lines (L1, L2 and L3), homozygous for transgene *TsVP*, were used in all of the experiments reported in this study.

### Generation of transgenic maize plants

*Agrobacterium tumefaciens* strain LBA4404, containing the pCAMBIA1300-Ubi-*TsVP-als* construct [9], was used to transform the maize (*Zea mays* L.) inbred line, DH4866. Agrobacterium-mediated maize transformation was performed as described by Li et al. [9]. Transgenic plants at the 3–4 leaf stage were selected by spraying the herbicide Lvhuanglong (containing 15% chlorsulphuron, Shenyang Pesticide Company, Shenyang, China) at 30 mg l^−1^ concentration. The PCR reaction was performed using specific primers for *TsVP*. Only the seeds from herbicide-resistant and PCR-positive plants were reserved. Plants were subsequently selected for two generations and then segregation analysis was carried out on seeds from independent self-pollinated T_1_ plants in order to select a homozygous T_2_ generation. Then southern blot analysis and a RT-PCR reaction were performed on the homozygous T_2_ lines to confirm the existence and expression of *TsVP*.

### PCR and Southern blot analysis

Genomic DNA was isolated from 0.1 g of young maize leaves by the cetyltrimethylammonium bromide (CTAB) method, PCR assays were performed as described by Li *et al.* [9]. The specific primers for the *TsVP* gene: 5′-CGACCTGTACGTCAGACACG-3′; 5′-ATGAACCACTGGAAGAGCGA-3′. To perform southern blot analysis, genomic DNA was extracted from 3 g of young maize leaves by the CTAB method. Genomic DNA (50 µg/sample) was digested overnight at 37°C with restriction enzyme *Bam*HI. The DNA restriction fragments were electrophoresed in 0.8% agarose gel, and then blotted to nylon membrane (Roche). PCR-amplified fragment containing full-length *TsVP* cDNA was used as a probe. Probe labeling and southern blotting were performed as described in the DIG System Manual (Roche).

### RNA isolation and RT-PCR

Total RNA was extracted from 0.1 g of root tips (1 cm segment of root apex's end) by TRIZOL (Sangon, Shanghai, China) and then treated with RNase-free DNase (Takara, Dalian, China). The DNase-treated RNA samples (500 ng) were used to synthesize cDNA using the RT Reagent Kit (TaKaRa, Dalian, China) according to the manufacturer's protocol, the RT reactions were performed at 42°C for 1.5 h using 0.5 µL of random hexamers. RT-PCR reactions were conducted as described by Li *et al.* [9]. The primers used for RT-PCR were 5′-TGATTCCTCCTGGTTGCC-3′ and 5′-GCTCTGATACACCCGCCTC-3′ for *TsVP*, were 5′-ATCACCATTGGGTCAGAAAGG-3′ and 5′-GTGCTGAGAGAAGCCAAAATAGAG-3′ for maize *actin1* gene.

### Plant growth and treatments under hydroponic conditions

The hydroponic cultures were created and maintained as described previously [25]. Maize seeds from the wild type and the homozygous transgenic lines were surface sterilized and germinated at 28°C in darkness for 3 days and then transferred into a nutrient solution containing sufficient phosphate (SP, 1,000 µM KH_2_PO_4_) to maintain healthy plants. After 20 days of growth, half of the seedlings continued to grow under SP conditions and the other half were cultured in a low phosphate (LP, 5 µM KH_2_PO_4_) nutrient solution for a further 25 days, after which the wild type plants and transgenic maize plants were collected for use in the following experiments: real-time RT-PCR, APase and vacuole H^+^-PPase assay and measurements of biomass, P content, root morphology and Pi uptake kinetic parameters. The nutrient solution contained the basal compositions: 2 mM Ca(NO_3_)_2_·4H_2_O, 1.25 mM NH_4_NO_3_, 0.1 mM KCl, 0.65 mM K_2_SO_4_, 0.65 mM MgSO_4_, 10.0 µM H_3_BO_3_, 0.5 µM (NH_4_)_6_Mo_7_O_24_, 1.0 µM MnSO_4_, 0.1 µM CuSO_4_·5H_2_O, 1.0 µM ZnSO_4_·7H_2_O, 0.1 mM Fe-EDTA. The initial pH value of nutrient solution was 6.0±0.1. Nutrient solution was replaced every 3 days.

### Real-time RT-PCR analysis

Total RNA from root tips was extracted and reverse transcribed as described in RNA isolation and RT-PCR. Real-time quantitative RT-PCR was performed on LightCycler 480 (Roche, Basel, Switzerland) using the SYBR Green I RT-PCR Kit (Takara, Dalian, China) according to the manufacturer's protocol. The primers used for Real-time RT-PCR were the same as in RT-PCR for *TsVP* and *actin1*, were 5′-CGCTGCTGTCCTTCCACTTC-3′ and 5′- GTCGCCGTACATGCCCTTGA-3′ for *ZmPIN1a*, were 5′-GCCGCTGCTGTCCTTCCACT-3′ and 5′- GTCGCCGTACATGCCCTTGA-3′ for *ZmPIN1b*, were 5′-CCCTCCTTCCACAACTACCG-3′ and 5′-CTTCTGAGGACGCCACATCG-3′ for AUX1 gene. The amplification conditions were as follows: 3 min at 95°C; 40 cycles of 10 s at 95°C, 30 s at 58°C. The relative expression levels were calculated with the 2^−ΔΔCT^ method [26], using maize *actin1* as an internal control.

### Vacuole H^+^-PPase assay

For vacuole H^+^-PPase assays, about 10 g of root tip fragments (1 cm segment from the root apex) were collected from wild type and transgenic maize plants grown under hydroponic conditions for 45 days as described above. Tonoplast vesicles were isolated by sucrose density gradient ultracentrifugation, as described previously [27]. H^+^-PPase hydrolytic activities were measured as the release of inorganic phosphate (Pi) according to the method used by Smart et al [28]. The Pi content was determined according to the method used by Lin and Morale [29]. Vacuole H^+^-PPase hydrolytic activity was presented as the difference in the measured values in the presence and absence of 50 mM KCl (K^+^-stimulated PPase activity).

### Measurement of biomass and P content

The wild type and transgenic plants were harvested after 45 days of growth in nutrient solution and washed in tap water for about 20 min. Roots and shoots were dried at 80°C in an oven to a constant weight. The plant dry weights were then recorded. To determine the P concentration, the roots and shoots were digested by the H_2_SO_4_–H_2_O_2_ method, the digested solution was diluted with double distilled water. The P concentration was determined as described by Murphy and Riley [30]. About 0.5 ml of diluted solution described above was added in 4 ml of coloration solution containing 0.6 M H_2_SO_4_, 0.5% (w/v) ammonium molybdate, 2% (w/v) ascorbic acid. The absorbance was read at 660 nm. P concentration values (mg P/g dry weight) represented the content of phosphorus that each gram of dry biomass contained. P content values (mg P per plant) represented the phosphorus content that different part of each plant contained, P content were calculated by multiplying the value of P concentration with the value of dry weight.

### Measurement of root morphology parameters

The number of axile root and lateral root were counted, total root length was measured with grid-line intersection method, root volume was determinated according to Musick et al. [31], total root absorption area and effective absorption area were measured using methyl blue method, and the average diameter of root was calculated from the formula: V = πR^2^L, where V stands for root volume, L for root length and R for average radius.

### Measurement of pH value

The hydroponic cultures and the basal compositions of nutrient solution were the same as in “Plant growth and treatments under hydroponic conditions”. The initial pH value of nutrient solution was 6.0±0.1. Nutrient solution was replaced every 3 days. Each of maize plants was cultured in 1 liter of nutrient solution. After 20 days of growth in sufficient phosphate (SP, 1,000 µM KH_2_PO_4_) nutrient solution, half of the seedlings continued to grow under SP conditions and the other half were cultured in a low phosphate (LP, 5 µM KH_2_PO_4_) nutrient solution for a further 25 days. After 10 days of culturing in different Pi conditions, the pH values of nutrient solution for wild type and transgenic plants were measured every day, using the portable pH meter (BANTE 2, BANTE, Shanghai, China).

### Estimation of Pi uptake kinetic parameters

Estimation of Pi uptake was carried out according to Drew and Saker [32]. The nutrient solution contained 50 µM Pi at the start. Pi uptake kinetic parameters were estimated according to Claassen and Barber [33].

### APase assay

To detect the acid phosphatase activity, different parts of maize plants, including root tip fragments (1 cm segment from the root apex), 1 cm fragments from the base of the roots and old leaves (the second leaf from the base of a seedling), were excised and ground to a powder under liquid nitrogen. Proteins were extracted as described by Li et al. [25]. The APase activity was determined according to Tadano and Sakai [34].

### Plant growth and treatments in flowerpots

Maize seeds from the wild type and transgenic homozygous lines were sown in flowerpots (diameter, 35 cm; height, 30 cm) in a naturally low-Pi soil (10 mg Pi kg^−1^ soil) with either no additional KH_2_PO_4_ (low Pi, LP) or mixed with 600 mg KH_2_PO_4_ (sufficient Pi, SP) per kilogram soil. Seedlings were allowed to grow under natural weather conditions until harvest (June to September) in Jinan, Shandong Province, and irrigated with tap water every 2 days. At the three-leaf stage, the plants were thinned to one plant per flowerpot. At various growth stages (the 20th, 40th, 60th and 80th day of growth), biomass, P content, gas exchange parameters and chlorophyll fluorescence were measured. When the plants reached flowering stage, the anthesis - silking interval (ASI) was recorded and only one ear was kept for each maize plant. At harvest time, the ear length, number of grain rows and grain number per row of mature ears were recorded; mature ears were dried to constant weight and then weighed to determine the grain yield per plant.

### Gas exchange and chlorophyll fluorescence measurement

To characterize photosynthetic performance, net photosynthesis, stomatal conductance and the intercellular CO_2_ content of maize plants grown in flowerpots were measured using a portable infrared gas analyzer-based open photosynthesis system (LI-6400, Li-Co Inc., Lincoln, NE, USA). All the measurements were carried out from 09:00 a.m to 10:30 a.m, when the ambient CO_2_ concentration was about 400 µmol mol^−1^ and air relative humidity was about 50%. The photosynthetic PFD was set at 800 µmol m^−2^ s^−1^, using an internal 6400-02B LED source, and the air flow rate was set at 600 µmol s^−1^. The instrument was stabilized according to manufacturer guidelines. Chlorophyll fluorescence was determined in intact plants using a pulse amplitude modulation fluorometer FMS-2 (Hansatech, Britain). The leaves previously selected for the measurement of gas exchange were used for the fluorescence measurements. Actual quantum yield of PSII (*Φ*_PSII_) was determined using natural light (about 800 µmol m^−2^ s^−1^) and a 1 s saturation pulse (4000 µmol m^−2^ s^−1^). After dark adaptation for 30 min, the potential maximum photochemical efficiency of PSII (Fv/Fm) was measured by application of a 1.6 s low (<0.1 µmol m^−2^ s^−1^) modulated light and a 1 s saturation pulse (4000 µmol m^−2^ s^−1^).

### Statistical analysis

All data were presented as the mean ± SD. Comparisons between transgenic and wild type plants were performed using Student's *t*-test. Value of *P*<0.05 was considered to be statistically significant. All statistical analyses were done using Sigma Plot 9.0.
